# Algae-Derived Polysaccharides Promote Growth Performance by Improving Antioxidant Capacity and Intestinal Barrier Function in Broiler Chickens

**DOI:** 10.3389/fvets.2020.601336

**Published:** 2020-12-02

**Authors:** Wen-Chao Liu, Yan Guo, Zhi-Hui Zhao, Rajesh Jha, Balamuralikrishnan Balasubramanian

**Affiliations:** ^1^Department of Animal Science, College of Coastal Agricultural Sciences, Guangdong Ocean University, Zhanjiang, China; ^2^Department of Human Nutrition, Food and Animal Sciences, College of Tropical Agriculture and Human Resources, University of Hawaii at Manoa, Honolulu, HI, United States; ^3^Department of Food Science and Biotechnology, College of Life Science, Sejong University, Seoul, South Korea

**Keywords:** algae-derived polysaccharides, antioxidant capacity, broilers, growth performance, gut health

## Abstract

This study aimed to determine the efficacy of dietary algae-derived polysaccharides (ADPs) from *Enteromorpha* on growth performance, intestinal morphology, intestinal permeability, and antioxidant capacity in serum, liver, and intestinal mucosa of broilers. Three hundred and ninety six day-old male chicks were randomly assigned to six dietary treatments containing 0 (Control), 1,000, 2,500, 4,000, 5,500, and 7,000 mg ADP/kg basal diet in a 35 day feeding trial. During day 1–21, compared with the control group, dietary 1,000–7,000 mg/kg ADP supplementation improved the average daily gain (ADG) and feed conversion ratio (*p* < 0.05). Overall (day 1–35), dietary inclusion of 1,000 mg/kg ADP increased the final body weight and ADG (*p* < 0.05). Besides, on day 21, dietary 2,500 mg/kg ADP supplementation increased the serum catalase (CAT) and liver total superoxide dismutase (T-SOD) activities (*p* < 0.05), whereas dietary 1,000–5,500 mg/kg ADP supplementation decreased malondialdehyde (MDA) contents in serum and liver (*p* < 0.05). On day 35, supplementation of 1,000 mg/kg ADP increased the serum glutathione peroxidase and CAT activities and liver T-SOD activities (*p* < 0.05). It decreased the MDA level of serum and liver (*p* < 0.05). Also, dietary 2,500 mg/kg ADP increased the villus height of jejunum and ileum on day 21 (*p* < 0.05), and dietary 4,000 mg/kg ADP increased the villus height of duodenum and ileum on day 35 (*p* < 0.05). On day 21, dietary 4,000 mg/kg ADP increased the CAT activities of the duodenum and T-SOD activities of jejunum and ileum and decreased the MDA contents in the duodenum, jejunum, and ileum (*p* < 0.05). On day 35, dietary inclusion of 1,000–7,000 mg/kg ADP reduced MDA contents of duodenum and jejunum (*p* < 0.05). Furthermore, dietary inclusion of ADP at 1,000–7,000 mg/kg decreased serum DAO activities at day 21 and day 35 (*p* < 0.05), and the serum D-lactic acid concentration was reduced by dietary supplementation of 1,000, 2,500, and 7,000 mg/kg ADP on day 21. In conclusion, dietary ADP exerted beneficial effects on growth performance, antioxidant capacity, and gut health in broilers; based on the studied parameters, the appropriate recommended dose is 1,000–4,000 mg/kg. These findings provided new insights into the potential application of ADP as natural growth promoters in broilers.

## Introduction

Due to restrictions or regulations on the use of antibiotic growth promoters in poultry production, there is an increasing demand for alternatives to antibiotic growth promoters like natural bioactive compounds to maintain the health and productivity of broilers (1–3). Natural polysaccharides, which are widely distributed in the microorganisms and plants, can improve an animal's gut health (4). Previously, numerous studies found that the natural polysaccharides derived from plants and fungus could be used as prebiotics to improve gut microflora balance and barrier function, ultimately promoting the growth of broiler chickens (5–8). Therefore, these natural and functional polysaccharides could replace antibiotics in the poultry industry. The marine ecosystem is an abundant reserve for natural polysaccharides. There are pieces of evidence supporting the biological and pharmacological role of polysaccharides in anti-carcinogenesis, antioxidation, anti-complementation, anti-inflammation, hematopoiesis, immunomodulation, and gastrointestinal tract protection (9, 10). Currently, marine polysaccharides have attracted considerable interest in the feed industry due to their biological activities (8, 11).

A naturally occurring marine green alga, *Enteromorpha*, is rich in polysaccharides. Many studies have reported multiple health-promoting benefits of *Enteromorpha* algae-derived polysaccharide (ADP) extract in anti-inflammation, against microbial pathogens, immunological regulation, and free-radical scavenging effects (12, 13). Also, broilers showed improved digestive function and immunity when fed with ADP from *Enteromorpha* (14, 15). Our recent study found that the ADP of *Enteromorpha* improves antioxidant performance and intestinal morphology in aged laying hens (16). However, to the best of our knowledge, the effects of ADP extracted from *Enteromorpha* on the antioxidant property and intestinal barrier function have not been evaluated in broilers yet. Hence, this study was conducted to evaluate the effects of dietary supplementation of ADP on growth performance, antioxidant property, and intestinal barrier function in broiler chickens.

## Materials and Methods

### Source of Algae-Derived Polysaccharides

The ADPs were extracted from the *Enteromorpha* by Qingdao Haida Biotechnology Co., Ltd. (Qingdao, China), with ≥48% purity, with a molecular weight of 4,929 Da. The ADPs are water-soluble sulfated polysaccharides obtained from the natural green alga *Enteromorpha* by enzymatic extraction, purification, concentration, and spray drying. Briefly, after crushing the algae, the algal powders are soaked in water. Then, the water extracts of the algae are subjected to stepwise enzymatic treatment with pectinase, cellulase, and papain. Then, the enzymes are inactivated, centrifugal concentrated, precipitated with ethanol, and finally spray dried to obtain the ADP used in this study. Based on the analysis by high-performance liquid chromatography, the polysaccharides mainly consisted of rhamnose (Rha), glucuronic acid (GlcA), glucose (Glc), galactose (Gal), and xylose (Xyl) monosaccharides. The molar percentages of monosaccharides in the ADP are as follows: Rha 40.6%, GlcA 9.3%, Glc 38.2%, Gal 5.6%, and Xyl 6.3%.

### Experimental Design, Birds, and Diets

For this study, 396 day-old male Arbor Acres broiler chicks (initial body weights 44.65 ± 0.56 g) were obtained from a commercial hatchery (Guangxi, China). The chicks were randomly allocated into one of six dietary treatments (6 replicate cages per treatment, with 11 broilers per cage) for a 35 day study period. Dietary treatments were as follows: basal diets supplemented with 0 (control group [CON]), 1,000, 2,500, 4,000, 5,500, and 7,000 mg ADP/kg basal diet. The basal diet was formulated (Table 1) to meet or exceed the nutrient requirements of National Research Council (17) of broilers in two phases—starter (1–21 days) and finisher (22–35 days); all diets were fed as in mash form. To ensure that the ADP was thoroughly mixed into the basal diet, ADP was first mixed with 1 kg of diet by hand, and then, the premix was mixed with the remaining diet by using a blender. The broilers were grown in a temperature-controlled room at 33 ± 1°C for the first 3 days and then gradually reduced by 3°C per week until reaching 24°C and maintaining humidity 65% for the rest of the study period. Stainless steel cages [90 (length) × 70 (width) × 40 (height) cm] with were used for housing. The birds had free access to feed and water.

**Table 1:** Composition and nutrient profile of basal diet (as-fed basis).

**Item**	**1–21 days**	**22–35 days**
**INGREDIENTS (g/kg)**		
Corn	572.0	607.4
Soybean meal (CP 45%)	292.4	250.3
Corn gluten meal (CP 60%)	44.0	38.3
Soybean oil	34.1	50.0
Limestone	9.1	10.2
Dicalcium phosphate	20.7	19.3
Salt	3.2	3.7
Methionine, 99%	3.3	3.7
Lysine-HCI, 24%	16.8	12.8
Threonine, 98.5%	1.8	1.8
Vitamin premix^a^	0.6	0.5
Trace mineral premix^b^	1.0	1.0
Choline, 50%	1.0	1.0
**CALCULATED NUTRIENT VALUES (g/kg)**		
Apparent metabolizable energy (MJ/kg)	12.64	13.39
Crude fat	63.12	74.89
Lys	14.93	12.10
Met	6.50	6.37
Met + Cys	13.72	14.13
Available phosphorus	5.15	4.60
**ANALYZED NUTRIENT VALUES (g/kg)**		
Gross energy (MJ/kg)	17.05	18.02
Crude protein	219.51	201.36
Ca	9.23	9.41
Total phosphorus	6.89	6.70

a *Provided per kilogram of diet: 15,000 IU of vitamin A, 3,750 IU of vitamin D_3_, 37.5 mg of vitamin E, 2.55 mg of vitamin K_3_, 3 mg of thiamin, 7.5 mg of riboflavin, 4.5 mg of vitamin B_6_, 24 μg of vitamin B_12_, 51 mg of niacin, 1.5 mg of folic acid, 0.2 mg of biotin, and 13.5 mg of pantothenic acid*.

b *Provided per kilogram of diet: 57.5 mg of Zn, 100 mg of Mn, 80 mg of Fe, 7.5 mg of Cu, 0.83 mg of I, and 0.5 mg of Se*.

### Sampling and Measurements

The broilers were weighed, and feed intake was recorded on day 1, 21, and 35. ADGs, average daily feed intake, and feed conversion ratio (FCR) were determined for each study phase. At the end of day 21 and 35, six birds (one bird from each replicate cage) were randomly selected from each treatment, and 3 ml blood samples per bird were collected from the wing vein by using a sterilized syringe with a needle (5 ml, 0.7 mm). The blood samples were then transferred into the vacuum tubes (5 ml, anticoagulant free, Xiangyuan Medical Ltd., Hebei, China). For serum analysis, the blood samples were centrifuged at 2,000 g at 4°C for 10 min to separate the serum and stored at −80°C to determine the antioxidant parameters and intestinal permeability biomarkers. The activities of the total superoxide dismutase (T-SOD), glutathione peroxidase (GSH-Px), and catalase (CAT), and the malondialdehyde (MDA) contents in serum were measured using commercial analysis kits (Jiancheng Bioengineering Institute, Nanjing, China) according to manufacturer's instructions. The levels of D-lactic acid and diamine oxidase (DAO) activities in serum were determined using enzyme-linked immunosorbent assay kits (96T, Yubo Biotechnology Co., Ltd. Shanghai, China) according to the manufacturer's instructions.

After blood sampling, the same birds were killed (by cutting carotid arteries) to collect the intestinal samples. Subsequently, the duodenum, jejunum, and ileum were separated, and ~2 cm segments of each at the middle location were collected and fixed in 10% buffered formalin until morphology analysis, stained with hematoxylin–eosin using standard histological techniques. The liver and remaining duodenum, jejunum, and ileum were rinsed in ice-cold phosphate-buffered saline (pH 7.0). Approximately 2 g liver tissues and mucosa of the duodenum, jejunum, and ileum samples were collected immediately for antioxidant parameter analysis. The activities of T-SOD, GSH-Px, CAT, and the MDA contents in the liver, mucosa of duodenum, jejunum, and ileum were measured using commercial kits (Jiancheng Bioengineering Institute, Nanjing, China) following manufacturer's instructions. Villus height, crypt depth, and villus width were measured at 40 × magnification using computer software (Olympus, DP72), then villus height-to-crypt depth ratio was calculated. The villus surface area (VSA) was calculated by using the formula (18):

$$\begin{array}{l} \text{VSA }=\;\left(2\text{π}\right)\;\times \;\left(\text{villus width}/2\right)\;\times \;\left(\text{villus height}\right) \end{array}$$

### Statistical Analysis

All the data were statistically analyzed using general linear model procedures of SAS (Statistical Analysis System, version 9.2, SAS Institute Inc., Cary, NC, USA) with cage as an experimental unit (19). Data were expressed as means; variability in data was expressed as the standard error of the mean. Orthogonal polynomial contrasts were used to test the linear and quadratic effects of the increasing levels of dietary ADP. The probability value of <0.05 was considered to be statistically significant, and 0.05 ≤ *p* < 0.10 was considered as a tendency.

## Results

### Growth Performance

The results of growth performance are presented in Table 2. During day 1–21, compared with the CON group, dietary supplementation of 1,000–7,000 mg/kg ADP improved the ADG and FCR (*p* < 0.05). During day 22–35, no significant differences were observed in ADG, average daily feed intake, and FCR among dietary treatments (*p* > 0.05). During the overall study period (day 1–35), dietary supplementation of 1,000 mg/kg ADP increased the final BW and ADG (*p* < 0.05), and supplementation of 1,000, 4,000, and 7,000 mg/kg ADP improved the FCR (*p* < 0.05).

**Table 2:** Effect of dietary supplementation with graded levels of algae-derived polysaccharides (ADP) on growth performance in broiler chickens.

**Items**	**Dietary ADP levels (mg/kg)**						**SEM**	***p*****-value**	
	**0**	**1,000**	**2,500**	**4,000**	**5,500**	**7,000**		**Linear**	**Quadratic**
Initial BW, g	44.04	45.33	45.10	45.21	44.07	44.27	0.56	0.204	0.308
Final BW, g	1,420.73^a^	1,560.42^b^	1,491.25^a,b^	1,479.50^a,b^	1,482.36^a,b^	1,500.19^a,b^	30.23	0.425	0.008
**1–21 DAYS**									
ADG, g	588.39^a^	657.16^b^	637.27^b^	627.53^b^	650.61^b^	648.62^b^	11.86	0.078	0.003
ADFI, g	1,092.67	1,088.83	1,072.67	1,078.30	1,081.35	1,075.50	10.46	0.174	0.620
FCR	1.861^a^	1.660^b^	1.685^b^	1.722^b^	1.666^b^	1.659^b^	0.035	0.020	0.003
**22–35 DAYS**									
ADG, g	786.65	854.73	810.64	805.69	785.80	802.85	25.81	0.911	0.170
ADFI, g	1,459.83	1,447.17	1,456.02	1,420.66	1,448.67	1,435.32	27.21	0.380	0.681
FCR	1.864	1.702	1.806	1.766	1.851	1.805	0.070	0.548	0.387
**1–35 DAYS**									
ADG, g	1,375.04^a^	1,511.89^b^	1,447.91^a,b^	1,433.21^a,b^	1,436.40^a,b^	1,451.46^a,b^	28.14	0.388	0.012
ADFI, g	2,552.50	2,536.08	2,528.68	2,499.00	2,530.10	2,510.83	24.71	0.141	0.792
FCR	1.862^a^	1.679^b^	1.750^a,b^	1.746^b^	1.769^a,b^	1.734^b^	0.039	0.124	0.031

*BW, body weight; ADG, average daily gain; ADFI, average daily feed intake; FCR, feed conversion ratio*.

*SEM, standard error of means*.

a,b *Means in the same row with different superscripts differ (P < 0.05)*.

### Antioxidant Status of Serum and Liver

As shown in Table 3, on day 21, dietary supplementation of ADP (2,500 mg/kg) increased the serum CAT activities (*p* < 0.05), whereas dietary supplementation of 1,000, 2,500, 4,000, and 5,500 mg/kg ADP decreased MDA contents in the serum (*p* < 0.05). Dietary supplementation of 2,500, 5,500, and 7,000 mg/kg ADP enhanced the liver T-SOD activities (*p* < 0.05), and supplementation of ADP at 1,000–7,000 mg/kg level reduced the liver MDA contents (*p* < 0.05). On day 35, addition of 1,000 mg/kg ADP increased the GSH-Px and CAT activities (*p* < 0.05) but decreased the MDA level (*p* < 0.05) in serum. Dietary supplementation of 1,000–7,000 mg/kg ADP enhanced the T-SOD activities (*p* < 0.05) but decreased the MDA contents (*p* < 0.05) in liver.

**Table 3:** Effect of dietary supplementation with graded levels of algae-derived polysaccharides (ADP) on antioxidant status of serum and liver in broiler chickens.

**Items**	**Dietary ADP levels (mg/kg)**						**SEM**	***p*****-value**	
	**0**	**1,000**	**2,500**	**4,000**	**5,500**	**7,000**		**Linear**	**Quadratic**
**DAY 21**									
**SERUM**									
T-SOD, U/ml	390.21	470.68	456.96	490.29	434.79	478.27	68.62	0.373	0.738
GSH-Px, U/0.1 ml	693.86	796.49	741.75	738.25	766.67	780.70	51.98	0.928	0.427
CAT, U/ml	6.71^a^	9.27^a,b^	10.22^b^	8.17^a,b^	8.11^a,b^	7.75^a,b^	1.01	0.265	0.045
MDA, nmol/ml	4.78^a^	3.11^b^	3.15^b^	3.20^b^	3.44^b^	3.77^a,b^	0.41	0.029	0.055
**LIVER**									
T-SOD, U/mg protein	262.08^a^	301.61^a,b^	330.94^b^	276.49^a,b^	314.17^b^	315.98^b^	17.05	0.364	0.020
GSH-Px, U/mg protein	31.31	32.93	40.73	34.57	43.30	38.19	7.65	0.619	0.622
CAT, U/mg protein	6.73	7.12	8.50	7.82	7.67	5.37	1.98	0.611	0.792
MDA, nmol/mg protein	0.79^a^	0.42^b^	0.46^b^	0.41^b^	0.46^b^	0.48^b^	0.060	0.002	0.024
**DAY 35**									
**SERUM**									
T-SOD, U/ml	449.24	556.39	549.33	582.66	527.16	570.64	62.74	0.191	0.569
GSH-Px, U/0.1 ml	607.62^a^	723.58^b^	662.18^a,b^	645.34^a,b^	693.76^a,b^	641.13^a,b^	34.81	0.746	0.086
CAT, U/ml	7.31^a^	11.54^b^	11.49^b^	9.77^b^	10.05^b^	10.00^b^	0.77	0.059	0.003
MDA, nmol/ml	5.56^a^	4.25^b^	4.23^b^	4.34^b^	4.58^a,b^	4.56^a,b^	0.34	0.035	0.058
**LIVER**									
T-SOD, U/mg protein	286.57^a^	339.43^b^	342.09^b^	357.65^b^	355.25^b^	357.13^b^	11.24	0.002	0.128
GSH-Px, U/mg protein	39.27	46.90	54.70	51.86	53.93	48.82	5.80	0.110	0.389
CAT, U/mg protein	6.48	7.17	7.89	5.87	7.72	5.08	1.40	0.863	0.354
MDA, nmol/mg protein	1.17^a^	0.86^b^	0.89^b^	0.85^b^	0.89^b^	0.81^b^	0.05	0.001	0.031

*T-SOD, total superoxide dismutase; GSH-Px, glutathione peroxidase; CAT, catalase; MDA, malondialdehyde*.

*SEM, standard error of the mean*.

a,b *Means in the same row with different superscripts differ (P < 0.05)*.

### Intestinal Morphology

As presented in Table 4, dietary supplementation of ADP at 4,000 and 5,000 mg/kg diet improved the villus height, villus width, and VSA of the duodenum (*p* < 0.05), dietary inclusion of 1,000, 2,500, 4,000, and 7,000 mg/kg ADP increased the villus height of jejunum (*p* < 0.05), and dietary inclusion of 2,500, 5,500, and 7,000 mg/kg ADP improved the villus height of ileum (*p* < 0.05) compared with control diet on day 21. Also, on day 35, dietary ADP increased the villus height and villus height-to-crypt depth ratio of the duodenum at 1,000, 4,000, and 7,000 mg/kg level and improved the VSA of the duodenum at 4,000 and 7,000 mg/kg diet level (*p* < 0.05). Dietary ADP supplementation improved the villus height of ileum at 2,500, 4,000, 5,500, and 7,000 mg/kg level and the VSA of ileum at 1,000, 2,500, 5,500 mg/kg diet level (*p* < 0.05).

**Table 4:** Effect of dietary supplementation with graded levels of algae-derived polysaccharides (ADP) on intestinal morphology in broiler chickens.

**Items**	**Dietary ADP levels (mg/kg)**						**SEM**	***p*****-value**	
	**0**	**1,000**	**2,500**	**4,000**	**5,500**	**7,000**		**Linear**	**Quadratic**
**DAY 21**									
**DUODENUM**									
Villus height, μm	430.68^a^	471.42^a,b^	464.00^a,b^	558.38^b^	559.75^b^	465.56^a,b^	26.80	0.004	0.335
Crypt depth, μm	101.80	113.03	108.49	100.89	135.71	103.32	17.75	0.928	0.600
Villus height-to-crypt depth ratio	4.59	4.65	4.36	5.72	4.82	4.82	0.66	0.309	0.337
Villus width, μm	61.55^a^	73.84^a,b^	77.29^a,b^	92.08^b^	89.96^b^	73.47^a,b^	7.71	0.011	0.873
Villus surface area, mm^2^	0.086^a^	0.110^a,b^	0.115^a,b^	0.162^b^	0.162^b^	0.108^a,b^	0.016	0.003	0.469
**JEJUNUM**									
Villus height, μm	369.93^a^	439.85^b^	450.57^b^	451.98^b^	437.79^a,b^	446.00^b^	23.87	0.024	0.164
Crypt depth, μm	94.509	91.327	101.058	108.95	107.66	100.51	9.91	0.243	0.581
Villus height-to-crypt depth ratio	4.26	4.98	4.56	4.30	4.20	4.61	0.48	0.891	0.324
Villus width, μm	68.40	80.61	91.99	79.40	73.51	76.19	10.33	0.346	0.242
Villus surface area, mm^2^	0.079^a^	0.110^a,b^	0.128^b^	0.111^a,b^	0.101^a,b^	0.108^a,b^	0.014	0.094	0.119
**ILEUM**									
Villus height, μm	226.84^a^	255.85^a,b^	302.26^b^	269.52^a,b^	302.03^b^	281.13^b^	18.42	0.044	0.106
Crypt depth, μm	64.86	59.35	77.08	69.52	75.04	75.91	6.97	0.319	0.884
Villus height-to-crypt depth ratio	3.77	4.50	4.07	4.08	4.25	3.79	0.50	0.833	0.475
Villus width, μm	62.64	71.88	60.30	63.69	60.33	60.19	6.55	0.776	0.660
Villus surface area, mm^2^	0.044	0.056	0.057	0.053	0.057	0.05	0.005	0.215	0.151
**Day 35**									
**DUODENUM**									
Villus height, μm	817.01^a^	896.55^b^	883.50^a,b^	945.72^b^	940.42^b^	893.72^b^	25.26	0.003	0.737
Crypt depth, μm	210.47	192.70	209.16	194.48	205.37	192.48	9.61	0.383	0.820
Villus height-to-crypt depth ratio	3.93^a^	4.75^b^	4.24^a,b^	4.88^b^	4.59^a,b^	4.68^b^	0.25	0.046	0.734
Villus width, μm	165.34	174.51	179.96	178.41	162.12	186.60	9.56	0.313	0.587
Villus surface area, mm^2^	0.423^a^	0.492^a,b^	0.504^a,b^	0.531^b^	0.480^a,b^	0.523^b^	0.031	0.025	0.507
**JEJUNUM**									
Villus height, μm	569.58	638.70	634.24	610.09	611.92	608.45	23.99	0.275	0.065
Crypt depth, μm	152.00	158.36	157.73	165.12	169.40	162.13	10.25	0.407	0.960
Villus height-to-crypt depth ratio	3.92	4.09	4.05	3.77	3.65	3.79	0.29	0.717	0.442
Villus width, μm	111.74	115.44	117.65	99.07	95.51	115.19	10.12	0.437	0.281
Villus surface area, mm^2^	0.200	0.231	0.232	0.189	0.182	0.223	0.021	0.723	0.084
**ILEUM**									
Villus height, μm	313.22^a^	360.21^a,b^	404.40^b^	372.62^b^	398.49^b^	373.01^b^	20.03	0.022	0.064
Crypt depth, μm	113.06	110.82	113.85	108.95	115.69	114.81	9.06	0.820	0.885
Villus height-to-crypt depth ratio	2.82	3.30	3.61	3.50	3.81	3.41	0.42	0.222	0.482
Villus width, μm	97.95	111.03	99.56	96.22	93.70	99.13	7.53	0.626	0.286
Villus surface area, mm^2^	0.093^a^	0.124^b^	0.126^b^	0.111^a,b^	0.117^b^	0.115^a,b^	0.008	0.115	0.007

*SEM, standard error of the mean*.

a,b *Means in the same row with different superscripts differ (P < 0.05)*.

### Antioxidant Capacity of the Intestinal Mucosa

On day 21 of the study, compared with the CON group, dietary 4,000 mg/kg increased the CAT activities, and 1,000–7,000 mg/kg ADP supplementation decreased the MDA contents in the duodenum (*p* < 0.05, Table 5). The inclusion of 4,000 mg/kg ADP in diet enhanced the T-SOD activities and reduced the MDA level of the jejunum (*p* < 0.05). Besides, dietary supplementation of ADP at 2,500, 4,000, 5,500, and 7,000 levels improved the T-SOD activities, 4,000, 5,500, and 7,000 mg/kg ADP improved the CAT activities, and 1,000–7,000 mg/kg ADP reduced the MDA contents in the ileum (*p* < 0.05). On day 35, dietary inclusion of 1,000–7,000 mg/kg ADP led to a reduction in MDA contents of the duodenum and jejunum in comparison with the CON group (*p* < 0.05). Dietary ADP at 1,000, 2,500, 4,000, and 7,000 mg/kg improved the T-SOD activities of jejunum (*p* < 0.05). Meanwhile, dietary addition of 5,500–7,000 mg/kg ADP decreased the MDA level of ileum (*p* < 0.05).

**Table 5:** Effect of dietary supplementation with graded levels of algae-derived polysaccharides (ADP) on antioxidant capacity of intestinal mucosa in broiler chickens.

**Items**	**Dietary ADP levels (mg/kg)**						**SEM**	***p*****-value**	
	**0**	**1,000**	**2,500**	**4,000**	**5,500**	**7,000**		**Linear**	**Quadratic**
**DAY 21**									
**DUODENUM**									
T-SOD, U/mg protein	381.87	393.15	370.01	338.40	389.18	328.59	58.34	0.569	0.721
GSH-PX, U/mg protein	54.99	70.08	61.58	63.53	64.42	66.85	11.78	0.752	0.589
CAT, U/mg protein	2.10^a^	3.94^a,b^	3.71^a,b^	4.60^b^	4.15^a,b^	4.28^a,b^	0.78	0.063	0.561
MDA, nmol/mg protein	0.69^a^	0.34^b^	0.31^b^	0.30^b^	0.32^b^	0.25^b^	0.09	0.014	0.085
**JEJUNUM**									
T-SOD, U/mg protein	246.96^a^	256.95^a,b^	267.23^a,b^	383.46^b^	293.19^a,b^	287.91^a,b^	40.44	0.043	0.218
GSH-PX, U/mg protein	67.18	58.11	58.5	34.867	74.317	39.75	16.86	0.229	0.675
CAT, U/mg protein	4.06	3.27	2.93	4.28	3.08	2.92	0.84	0.931	0.233
MDA, nmol/mg protein	0.52^a^	0.31^a,b^	0.38^a,b^	0.21^b^	0.30^a,b^	0.28^a,b^	0.09	0.049	0.855
**ILEUM**									
T-SOD, U/mg protein	384.33^a^	501.31^a,b^	513.09^b^	508.04^b^	502.15^b^	538.22^b^	37.11	0.044	0.131
GSH-PX, U/mg protein	147.34	149.09	153.93	155.87	156.77	159.19	12.62	0.571	0.994
CAT, U/mg protein	2.99^a^	4.75^a,b^	4.83^a,b^	5.96^b^	5.67^b^	5.63^b^	0.81	0.015	0.702
MDA, nmol/mg protein	0.98^a^	0.63^b^	0.54^b^	0.50^b^	0.49^b^	0.44^b^	0.10	0.005	0.156
**DAY 35**									
**DUODENUM**									
T-SOD, U/mg protein	276.22	283.42	320.05	274.41	228.58	263.61	48.50	0.888	0.598
GSH-PX, U/mg protein	78.03	78.84	64.52	72.29	70.99	65.02	13.16	0.604	0.797
CAT, U/mg protein	6.86	6.24	7.52	6.12	6.79	6.01	1.65	0.900	0.820
MDA, nmol/mg protein	0.44^a^	0.23^b^	0.21^b^	0.27^b^	0.19^b^	0.18^b^	0.05	0.052	0.040
**JEJUNUM**									
T-SOD, U/mg protein	238.22^a^	320.19^b, c^	297.51^b^	336.68^b, c^	254.03^a^	348.12^c^	13.76	0.001	0.151
GSH-PX, U/mg protein	94.78	91.55	105.74	122.11	106.70	115.73	19.388	0.264	0.742
CAT, U/mg protein	3.92	4.06	7.04	4.94	4.98	5.08	1.22	0.294	0.378
MDA, nmol/mg protein	0.79^a^	0.72^a,b^	0.55^a, b, c^	0.52^a, b, c^	0.41^b^	0.33^c^	0.11	0.071	0.856
**ILEUM**									
T-SOD, U/mg protein	365.68	387.94	380.90	433.59	377.15	342.12	44.97	0.582	0.532
GSH-PX, U/mg protein	87.73	83.80	98.10	91.55	90.26	84.29	8.92	0.945	0.887
CAT, U/mg protein	7.73	7.25	7.20	8.60	7.87	7.08	1.84	0.975	0.786
MDA, nmol/mg protein	0.65^a^	0.50^a,b^	0.51^a,b^	0.50^a,b^	0.46^b^	0.48^b^	0.05	0.075	0.180

*T-SOD, total superoxide dismutase; GSH-Px, glutathione peroxidase; CAT, catalase; MDA, malondialdehyde*.

*SEM, standard error of the mean*.

a−c *Means in the same row with different superscripts differ (P < 0.05)*.

### Serum Markers of Intestinal Permeability

The effects of dietary ADP on intestinal permeability biomarkers are presented in Table 6. Dietary inclusion of ADP at 1,000–7,000 mg/kg decreased serum DAO activities on day 21 and 35 (*p* < 0.05). Also, the serum D-lactic acid concentration was reduced by dietary supplementation of 1,000, 2,500, and 7,000 mg/kg ADP on day 21.

**Table 6:** Effect of dietary supplementation with graded levels of algae-derived polysaccharides (ADP) on serum markers of intestinal permeability in broiler chickens.

**Items**	**Dietary ADP levels (mg/kg)**						**SEM**	***p*****-value**	
	**0**	**1,000**	**2,500**	**4,000**	**5,500**	**7,000**		**Linear**	**Quadratic**
**DAY 21**									
D-Lactic acid, ng/ml	6.07^a^	4.36^b^	4.49^b^	4.81^a,b^	4.94^a,b^	4.77^b^	0.41	0.071	0.031
DAO, U/ml	32.72^a^	28.25^b^	27.03^b^	28.15^b^	27.13^b^	28.19^b^	1.40	0.039	0.075
**DAY 35**									
D-Lactic acid, ng/ml	3.77	3.14	2.74	3.06	3.20	3.35	0.54	0.320	0.401
DAO, U/ml	25.80^a^	21.35^b^	21.79^b^	21.55^b^	22.87^b^	20.28^b^	0.91	0.013	0.043

*DAO, diamine oxidase*.

*SEM, standard error of means*.

a,b *Means in the same row with different superscripts differ (P < 0.05)*.

## Discussion

### Growth Performance

In recent years, utilizing polysaccharide compounds in the feed as prebiotics and growth promoters are at the center of attention in the poultry industry. The functional polysaccharides are non-digestible ingredients due to the presence of β-1,4 linkages (11, 16). Consumption of functional polysaccharides from medicinal plants and fungus could modulate the gut microbiota and improve health (3), thereby improving the growth performance in broilers (5–7, 20). The findings of the present study indicated that the inclusion of dietary ADP could promote the growth performance of broiler chickens. Similar to our results, Li et al. (21) observed that the dietary ADP of *Enteromorpha* at the inclusion rate of 0.5% improved the ADG and FCR of broilers through enhancing immune function. Similarly, other sources of marine polysaccharides, such as chitooligosaccharide (COS), have also been reported to improve the growth rate in broilers. According to Li et al. (22), broilers fed diet that contained 30 mg/kg COS showed higher FCR when compared with control. Likewise, Huang et al. (23) and Li et al. (24) found that the dietary inclusion of 150 or 100 mg/kg COS improved ADG and FCR in broilers. A wide range of mechanisms can underlay to decipher the beneficial effects of these natural marine polysaccharides on enhancing the growth performances, antioxidant capacity, and intestinal health status (22), promoting the growth of beneficial gut microbiota (8), immunity (14, 21), and nutrient digestibility (15) in broilers. However, Biggs et al. (25) and Shang et al. (6) observed no significant difference in the growth performance of broiler chickens fed a diet containing natural polysaccharides. The variation in response to natural dietary polysaccharides in different trials could be attributed to the polymerization levels, purity, supplemental dosage, experimental period and conditions, and the source of polysaccharides. Besides, it was remarkable that the positive effects of ADP on growth performance were mainly manifested during the starter phase (day 1–21) in this study. It was probably due to external stressors leading to altered physiology and weak immunity in broilers (22). The functional polysaccharides could improve the physiological function and immune function against a variety of stressors, subsequently stimulating the growth rate of broilers.

### Antioxidant Status of Serum and Liver

With the ongoing intensification of the poultry industry, broiler chickens are continuously exposed to various external stressors resulting in excess production of reactive oxygen species (ROS) and disturbing the redox balance in the chicken's body, contributing to oxidative stress (26, 27). Oxidative stress is one of the major factors adversely influencing the performance of chickens and leads to many metabolic disorders (16, 28, 29). Antioxidant enzymes, such as SOD and CAT, are known for their scavenging oxygen free radical property, thereby providing the first line of cellular defense against oxidative damage (30, 31). As an end product from lipid peroxidation, the MDA is used as a biomarker to measure the level of oxidative stress (32). The results of this study showed that the dietary ADP supplementation elevates the SOD activities of liver and CAT activities of serum but reduced the MDA levels in serum and liver. Duan et al. (13) provided direct evidence about the antioxidant properties of *Enteromorpha* ADP using *in vitro* studies. However, scientific information from the *in vivo* studies on the antioxidant capacity of ADP from *Enteromorpha* in broilers is relatively scarce. Due to the antioxidant bioactivities, the natural polysaccharides have received broad interest in animal nutrition, and several researchers have evaluated the antioxidant effects of marine polysaccharides in poultry and suggested that the antioxidant activity of marine polysaccharides may be due to the hemiacetal hydroxyl in the structure, which can scavenge the free radicals (9). Park et al. (33) reported that the inclusion of COS in drinking water could enhance the antioxidant capacity of broilers, and the present study found that the ADP supplementation improves serum SOD and liver CAT activities with a reduction in serum and liver MDA contents, which is similar to the finding in laying hens by Guo et al. (16). Similarly, Long et al. (34) reported that the dietary addition of 2,000 mg/kg *Lycium barbarum* polysaccharides improved SOD and GSH-Px activities with decreased MDA concentration in the serum and liver of broilers on day 42. It can be presumed that the improved antioxidant capacity observed in the present study is primarily attributed to the antioxidant characteristics of ADP (35). Thus, the marine polysaccharide ADP might be used as an ideal natural antioxidant agent in broilers due to synthetic antioxidants' toxicity. Also, improved antioxidant capacity might be closely linked with the enhanced growth performance in this study.

### Intestinal Barrier Function

The gut is the center for digestion and absorption in animals. Additionally, it is known to function as an intestinal barrier, intestinal structure, antioxidant status, and permeability and play a critical role in nutrient processing and enhancing animal performance (4, 16). In this study, dietary supplementation of graded dosages of ADP exerted beneficial effects on intestinal morphology in villus height in both the duodenum and ileum. Similar to our results, Li et al. (22) found that dietary 30 mg/kg COS exerted a positive effect on duodenal morphology in broilers, as evidenced by the increased villus height and the ratio of villus height to crypt depth. Shang et al. (6) also reported that the natural dietary 0.5% polysaccharides of fructo-oligosaccharides could improve the villus height and total mucosa thickness of the ileum in broiler chickens on day 21. Similarly, Berrocoso et al. (36) reported increased villus height and villus height:crypt depth ratio when broilers were fed *in ovo* with raffinose as a prebiotic. Also, Abolfathi et al. (27) reported that dietary 1,000 mg/kg extract of elecampane (*Inula helenium L*.) rhizome that contains polysaccharide compounds increase villus height and villus height:crypt depth ratio of jejunum and ileum in broilers. These favorable alterations in intestinal morphology might be due to the prebiotic function of polysaccharides that could work as a substrate for the intestinal microflora and aids in stimulating the fermentation rate and increasing the production of short-chain fatty acids. The short-chain fatty acid benefits the differentiation and proliferation of intestinal epithelial cells (37). Similarly, other researchers suggest a positive association between intestinal morphological and antioxidant capacity in broilers (38–40), which was seen in our study as well.

The intestinal mucosa acts as a barrier to the external environment. Also, it is vulnerable to oxidative damage due to the massive workload and high rate of oxidative metabolism of the intestine, which results in the abundant release of ROS (28). Therefore, the intestine is a key source of ROS and particularly sensitive to oxidative stress compared with other tissues (41). Our current study's findings indicate that the basal diet supplemented with ADP enhances antioxidant enzyme (SOD, CAT) activities and successfully inhibits lipid peroxidation as observed in the decrease of MDA content in the intestinal mucosa. The results strongly agree with the available literature on natural polysaccharides. For instance, the inclusion of 30 or 350 mg/kg COS in broilers' diet was reported to improve the intestinal mucosa oxidative damage by enhancing SOD and GSH-Px enzyme activities and reducing the content of MDA (22, 42). The *Artemisia argyi aqueous* extract that contains an abundant amount of natural polysaccharides, feeding to broilers at 500–2,000 mg/kg level, increased the total antioxidant capacity, SOD, and GSH-Px activities by decreasing the MDA levels in small intestinal tissues (43). Thus, the natural polysaccharides can act as a defensive mechanism for antioxidant status in broilers' intestinal mucosa and can prevent the uncontrolled formation of ROS and/or directly scavenging the free radicals.

Serum D-lactic acid levels and DAO activity levels are considered sensitive biomarkers for intestinal permeability and generally determined to examine the extent of the damage or repair in the intestinal tract (44). An increase in serum D-lactic acid levels reflects an increased intestinal permeability (45). The DAO is secreted from the epithelial cells of the intestine and known for its hyper-endocellular enzymatic activity. The presence of higher DAO levels in serum indicates elevated functioning of intestinal barrier and permeability (44). In the present study, both D-lactic acid levels and DAO activities were significantly decreased by the inclusion of ADP in the broiler's diets, which indicated that the ADP improved the intestinal permeability and ultimately protected the intestinal barrier function. Similarly, previous literature showed that dietary supplementation of marine polysaccharides of 30 mg/kg COS resulted in a lower serum DAO activity and tended to reduce the concentration of D-lactic acid in broilers (22). The beneficial effects of ADPs on intestinal permeability might be related to improvements in tight junction protein expression of the intestine; however, the specific mechanism warrants further investigations. Coupled with the findings of higher antioxidant capacity of the intestinal epithelium along with improved morphological characteristics and enhanced intestinal barrier function observed in our study, it could provide the right consistency in improving the growth performance of broilers. The assessment of D-lactic acid and DAO can be performed in blood, which is a less invasive method. However, additional studies are required to recommend it for practical applications.

## Conclusion

**Figure 1:**
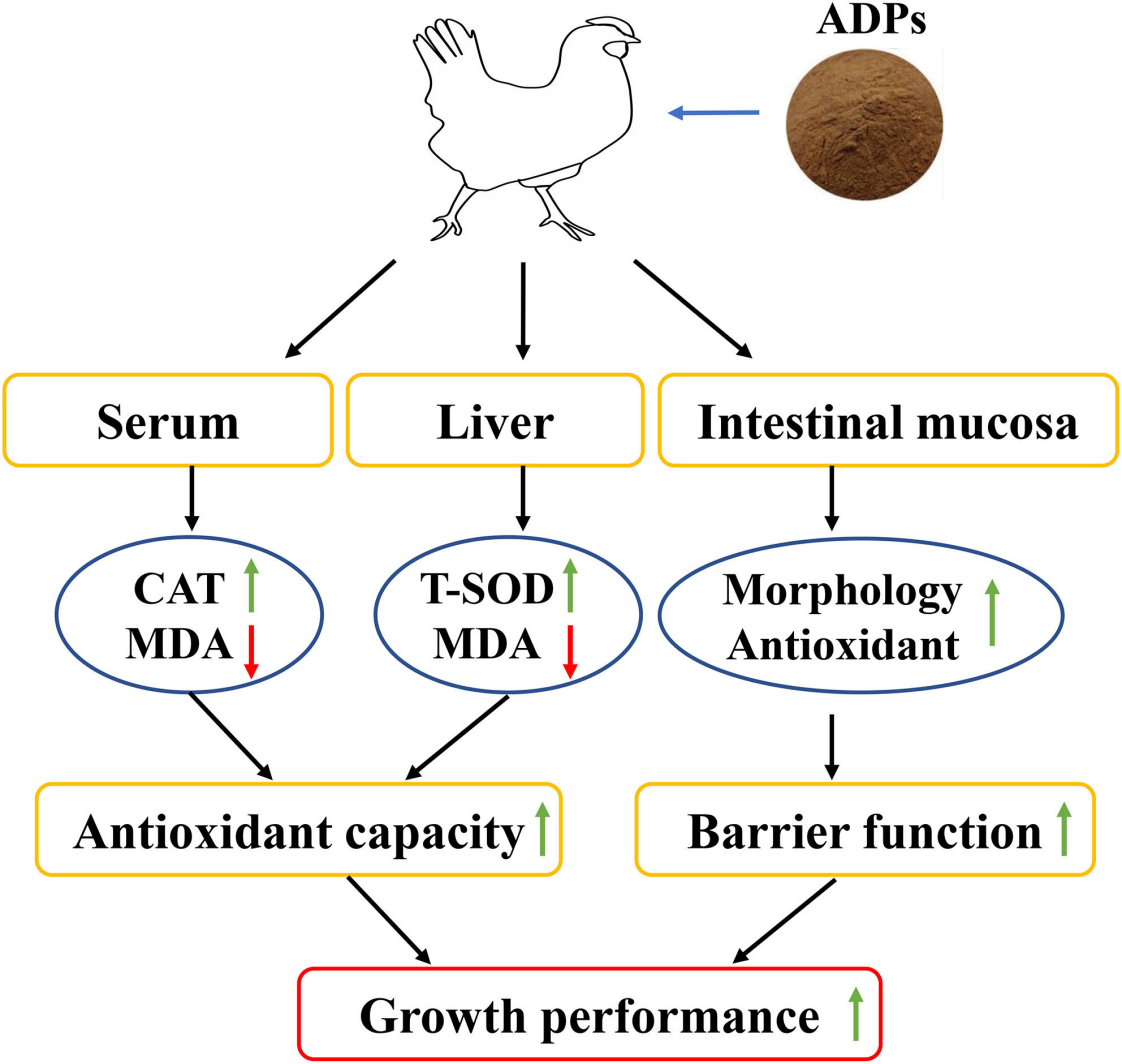
Schematic diagram showing how Algae-derived polysaccharides (ADP) promote broiler chicken's growth performance by improving antioxidant capacity and intestinal barrier function.

Our study shows that the dietary inclusion of ADP could promote growth performance, enhance the antioxidant property, and improve intestinal barrier function in terms of morphology, antioxidant status, and permeability of the intestinal mucosa (Figure 1). According to the study results, supplementation levels could be recommended at 1,000–4,000 mg/kg ADP in the broilers' diet. These findings provided a reference and new insights on dietary inclusion of ADP to promote growth and natural antioxidants, thus reducing the usage of antibiotics and synthetic antioxidants in broiler nutrition.

## Data Availability Statement

The original contributions presented in the study are included in the article/supplementary materials, further inquiries can be directed to the corresponding author/s.

## Ethics Statement

The animal study was reviewed and approved by Animal Care Committee, Guangdong Ocean University.

## Author Contributions

W-CL, RJ, and BB: conceptualization. W-CL: methodology. W-CL and YG: analysis and data curation. W-CL and BB: writing-original draft preparation. RJ and BB: writing-review and editing. RJ: supervision. W-CL and Z-HZ: project administration. W-CL: funding acquisition. All authors contributed to the article and approved the submitted version.

## Conflict of Interest

The authors declare that the research was conducted in the absence of any commercial or financial relationships that could be construed as a potential conflict of interest.
